# Disagreement in physical activity assessed by accelerometer and self-report in subgroups of age, gender, education and weight status

**DOI:** 10.1186/1479-5868-6-17

**Published:** 2009-03-25

**Authors:** Sander M Slootmaker, Albertine J Schuit, Marijke JM Chinapaw, Jacob C Seidell, Willem van Mechelen

**Affiliations:** 1 EMGO Institute, Department of Public and Occupational Health, Body@Work Research Center Physical Activity, Work and Health, TNO-VU University Medical Center, Amsterdam, The Netherlands; 2 National Institute for Public Health and the Environment, Bilthoven, The Netherlands; 3 Institute of Health Science, Faculty of Earth and Life Sciences, VU University Amsterdam, The Netherlands

## Abstract

**Background:**

The purpose of this study is to compare self-reported time (by questionnaire) and objectively measured time (by accelerometer) spent on physical activity at moderate (MPA) and vigorous intensity (VPA) in subgroups of age, gender, education and weight status.

**Methods:**

In total, 236 adolescents (aged 12–18) and 301 adults (aged 22–40), completed the questionnaire and wore an accelerometer for two weeks.

**Results:**

Adolescents reported exceptionally more time spent on MPA (mean difference 596 ± 704 min/wk) and VPA (mean difference 178 ± 315 min/wk) than was assessed objectively by the accelerometer. Based on the questionnaire, high educated adolescents spent more time on MPA (205 min/wk, *p *= 0.002) and VPA (120 min/wk, *p *= 0.01) than low educated adolescents, but according to the accelerometer they spent less time on MPA (149 min/wk, *p *= 0.001) and VPA (47 min/wk, *p *= 0.001). Among adults there was moderate agreement between self-reported time and objectively measured time spent on MPA, but in general the reported time spent on MPA (mean difference 107 ± 334 min/wk) and VPA (mean difference 169 ± 250 min/wk) exceeded the time measured with the accelerometer. Overweight adults reported significantly more VPA (57 min/wk, *p *= 0.04) than normal weight adults, but this was not confirmed by the accelerometer data.

**Conclusion:**

We observed large differences in time spent on MPA and VPA measured by questionnaire and accelerometer in adolescents but reasonably good agreement in adults. Differences between methods varied by gender, education and weight status. This finding raises serious questions about the use of questionnaires to quantify MPA and VPA in adolescents. There is a clear need in advanced valid assessments of PA in adolescents.

**Trial number:**

ISRCTN93896459

## Background

On a population level there are great differences in physical activity (PA) between groups. Studies that used self-report assessment of PA have consistently found that women are less physically active than men, overweight subjects are less active than normal weight subjects, and adults are less active than adolescents [1-8]. These findings have been confirmed in studies using objective assessment of PA (i.e. accelerometry) [9-12]. Yet, there is considerable disagreement between studies in the magnitude of these differences. Physical activity questionnaires have been shown to report smaller differences between gender and ethnic subgroups compared to accelerometers [13]. This disagreement will partly reflect real differences but may also be partly attributable to the method used to measure PA.

Self-administered questionnaires assessing PA is the most commonly used method of assessing PA, because it is relatively inexpensive and easy to use in large-scale studies. However, self-reported levels of PA are based on the persons' perception of his or her own quantity of PA and is therefore prone to misinterpretation, social desirability and reliant on accurate recall of the type, intensity, frequency and duration of daily (in)activities [14-16]. A few studies have indicated that overestimation of PA is greater in adolescents and subjects with higher body fatness [17-20].

Waist-mounted accelerometers provide a more objective estimate of PA within a given day, or over several days by measuring bodily accelerations at the hip [21]. They offer real time data storage, which is a distinct advantage over self-report methods. However, they do not provide qualitative information on the type of activity.

Accuracy of the accelerometer is dependent on the type of activity, with little concordance between accelerometry and energy expenditure during movements with static hip position (e.g. lifting objects and cycling), and the best concordance between accelerometry and energy expenditure for walking activities [22,23]. There are several types of accelerometers available, i.e. single axis and multiple axis accelerometers (e.g. tri-axial). These accelerometers have been used in previous studies among adolescents and adults in free-living situations and are feasible in large studies [24-27]. The feasibility of using accelerometers in large studies depends on the financial means to purchase large amounts of accelerometers and the available time and manpower to process the data.

The purpose of this study was twofold. The first aim was to compare time spent on moderate and vigorous intensity PA according to an objective measure (accelerometer) with time spent according to a self-administered measure (PA questionnaire). The second aim was to examine if there is differentiation in reporting PA levels versus objectively measured PA among subgroups of age, gender, education and weight status.

## Methods

### Participants

A total of 286 adolescents (aged 12 to 18 years) and 332 young adults, (aged 22 to 40 years) with different educational levels were recruited in the surroundings of Amsterdam, the Netherlands. Five secondary schools and eight worksites facilitated the recruitment and measurements for this study. Inclusion criteria were ability to walk (without aid) and not being pregnant. The young adults consisted mainly of office workers. This study is part of the PAM project, in which among other things the effectiveness of providing the PAM accelerometer (PAM. B.V., Doorwerth, the Netherlands) in combination with an individually tailored PA advice is evaluated in relatively inactive adolescents and young adults. The PAM project is described in more detail elsewhere [28]. The study protocol of the PAM project was approved by the Medical Ethics Committee of the VU University Medical Center. All participants and their parents gave their informed consent.

### Procedures

Body height and weight were obtained on the first day of measurement. Body weight was measured in light clothing without shoes to the nearest 0.2 kg, using a digital balance (model Seca 888, Seca GmbH & Co, Hamburg, Germany). Body height was measured to the nearest 0.1 cm with a stadiometer (model Seca 225, Seca GmbH & Co, Hamburg, Germany). Educational level and date of birth were assessed by questionnaire. The participants received an accelerometer together with written and verbal instructions and a practical demonstration on how to wear the accelerometer. For practical considerations, the participants were asked to wear the accelerometer for fourteen consecutive days during waking hours. After this period the participants completed a short PA questionnaire and were asked if they had worn the accelerometer during sports. Participants who completed all measurements were given a small incentive.

### Assessment of Physical Activity

#### Physical Activity Questionnaire

The Activity Questionnaire for Adolescents & Adults (AQuAA, additional file 1) is a short questionnaire, developed to assess PA and sedentary behavior at work, at school, during leisure time and transport. The structure of the AQuAA is based on the SQUASH-questionnaire [29] with two adaptations made. First, the AQuAA also registers time spent on light intensity activities and sedentary behaviors whereas the SQUASH does not. Second, the questions in the AQuAA refer to activities performed in the previous seven days and the SQUASH refers to an average week in the past few months. Based on the assumption that one sleeps 8 hours per day, sixteen hours (960 minutes) was considered the maximum amount of time per day a person can spend on PA. Five (2%) adolescents and three (1%) adults were excluded because they exceeded this maximum time.

Unpublished data of a validation study (Chinapaw, MJ, Slootmaker, SM, Zuidam, M, Schuit, AJ, and van Mechelen, W. Test-Retest Reproducibility and Validity of the Activity Questionnaire for Adults and Adolescents, submitted) show that the test-retest reliability of the AQuAA was moderate in both adolescents (intra-class correlations; ICCs ranging from 0.30 to 0.59) and adults (ICCs ranging from 0.49 to 0.60). Spearman correlation coefficients between the time spent on sedentary, light, moderate and vigorous activities compared to the MTI Actigraph accelerometer were low and non-significant for adolescents (0.23, 0.11, -0.21 and 0.21 respectively) and for adults (0.15, 0.07, -0.06 and 0.12 respectively).

#### Accelerometer

The PAM accelerometer (PAM, model AM101, PAM B.V., Doorwerth, The Netherlands) is an uni-axial accelerometer that can be easily clipped to a belt or waistband. The PAM converts vertical accelerations by a piezoelectric sensor into an activity score, i.e. the PAM score. The PAM also offers the possibility to monitor the number of minutes spent on moderate (MPA) and vigorous intensity physical activity (VPA) per day. PAM data can be easily downloaded via a docking station to a personal computer. The validity of the PAM accelerometer has been tested in a laboratory setting and has shown results to be similar to the MTI Actigraph for estimating energy expenditure in walking and stair walking [30]. The PAM shows high reliability (ICC = 0.80) at a frequency of 3 Hz. Before the study, all PAMs were calibrated on a shaking machine at 3 Hz and thresholds for MPA and VPA were set-up by using the manufacturer equation (PAM score = (MET*0.9–1)*100).

The participants were asked to wear the accelerometer always on the right hip (sagittal line) for 14 days during waking hours, accept during water sports and bathing because the PAM is not waterproof. During the fourteen days the PAM registered, the PAM scores were not visible for the participants in order to minimize the influence of wearing the accelerometer. Due to technical problems, accelerometer data could not be read of 23 (8%) adolescents and 13 (4%) adults. Daily PAM scores below five were converted to missing values because this is an indication that the PAM was not worn. Eighteen adolescents (7%) and 7 adults (2%) did not wear the accelerometer for at least three days in the last week and were therefore excluded. As a consequence, 236 adolescents and 301 adults were included in the analyses.

### Data analysis

Educational level was categorized into low and high educational level, according to the Dutch educational system. A high educational level comprised of secondary schools preparing for college or university. All other levels of education were defined as low educational level. BMI (kg/m^2^) was used to categorize the participants as either normal weight or overweight. Adolescents were categorized as being overweight based on the gender and age specific Cole criteria [31]. Adults were defined as being overweight when their BMI was ≥ 25 kg/m^2^.

For the purpose of this study, cut off points for MPA (range for adolescents, 5–8 metabolic equivalents, MET, and adults 4–6.5 MET) and VPA (adolescents, > 8 MET and adults > 6.5 MET) were used based on the Dutch Public Health Physical Activity recommendation [32]. The minutes spent in MPA and VPA per day registered by the accelerometer were averaged over the number of days the participant had worn the accelerometer in the last week. This average was multiplied by seven days. Minutes per week of MPA, VPA and MVPA from the questionnaire were calculated by frequency times duration of the activities.

Bicycling is an important mode of transportation in the Netherlands but poorly registered by the accelerometer. Therefore the agreement between the minutes registered by the accelerometer on MVPA were compared to AQuAA data with and without the inclusion of self-reported minutes of cycling.

Because the distribution of self-reported and objectively measured MPA, VPA and MVPA was skewed, we presented data as medians and inter-quartile ranges, per age-group. We used non-parametric tests to test differences in questionnaire and the accelerometer, and to test the differences within the subgroups of gender, educational level and weight status.

In a multivariate regression model gender, educational level, BMI, and age were added as independent variables and disagreement between both methods (AQuAA minus PAM) was the dependent variable. Bland-Altman plots with 95% limits of agreement were calculated as the main measures of agreement between (and within) the instruments. Statistical significance was set at p < 0.05.

## Results

### Characteristics of the study population

**Figure 1:**
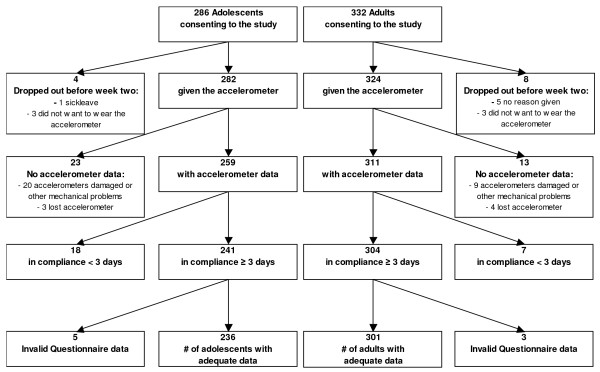
**The flowcharts of the exclusion of recruited adolescents and adults**.

Figure 1 represents the flowcharts of the exclusion of recruited adolescents and adults in the study. The excluded subjects did not differ significantly from the final sample with respect to gender, education and weight status. The average age of the adolescents and adults was 15 (SD = 1) and 31 (SD = 3) years, respectively. Sample characteristics are shown in table 1. Ninety-six adolescents (41%) and 158 adults (52%) have worn the accelerometer for seven consecutive days in the last week.

**Table 1:** Sample Characteristics

Variable	Adolescents	Adults
Female	142 (60%)	169 (56%)
Higher education	163 (69%)	206 (68%)
Overweight	21 (9%)	112 (37%)

### Agreement between reported and objectively measured physical activity level

**Figure 2:**
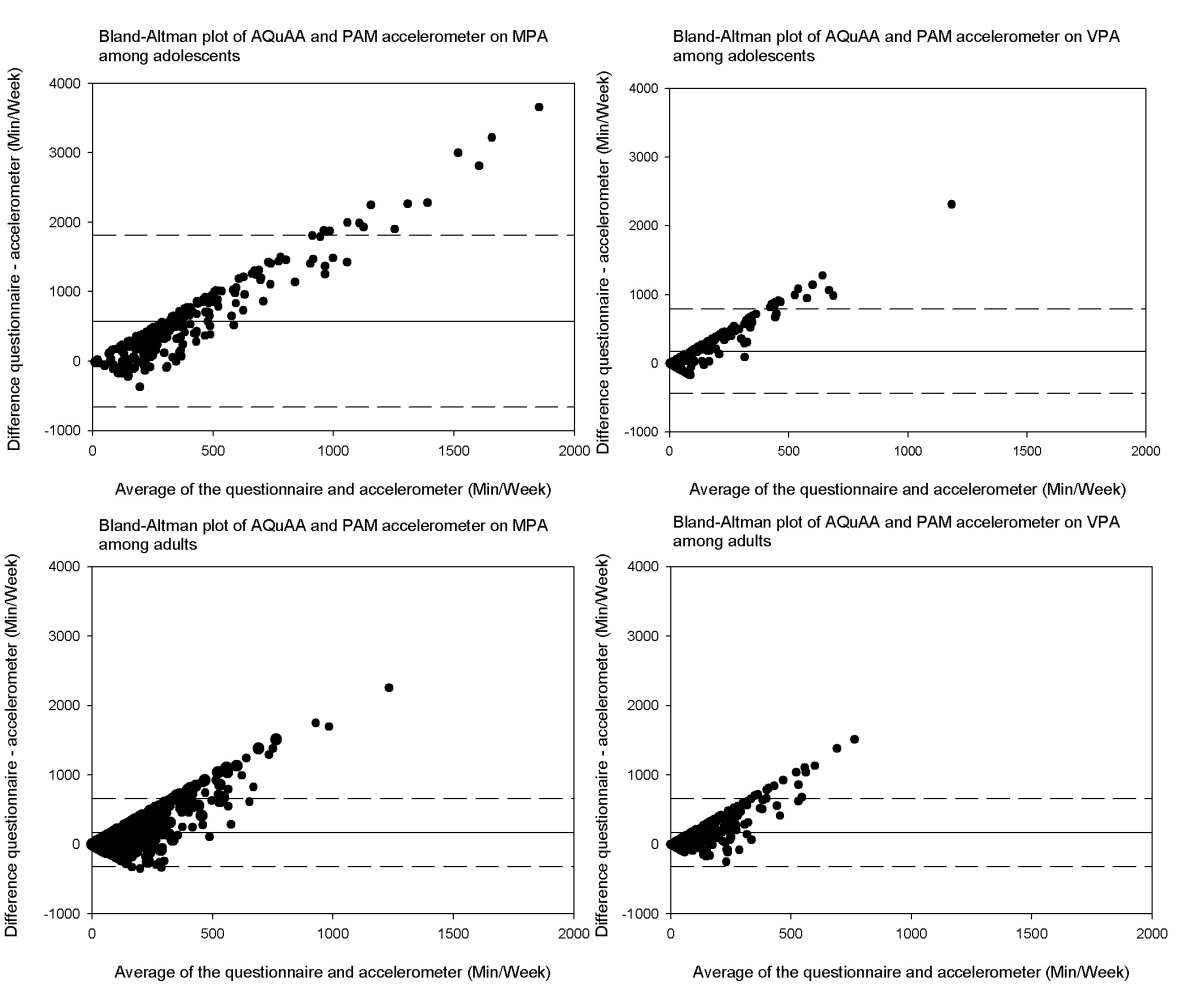
**Bland-Altman plots of moderate and vigorous physical activity (min/wk) assessed by AQuAA and PAM accelerometer among adolescents and adults**.

**Figure 3:**
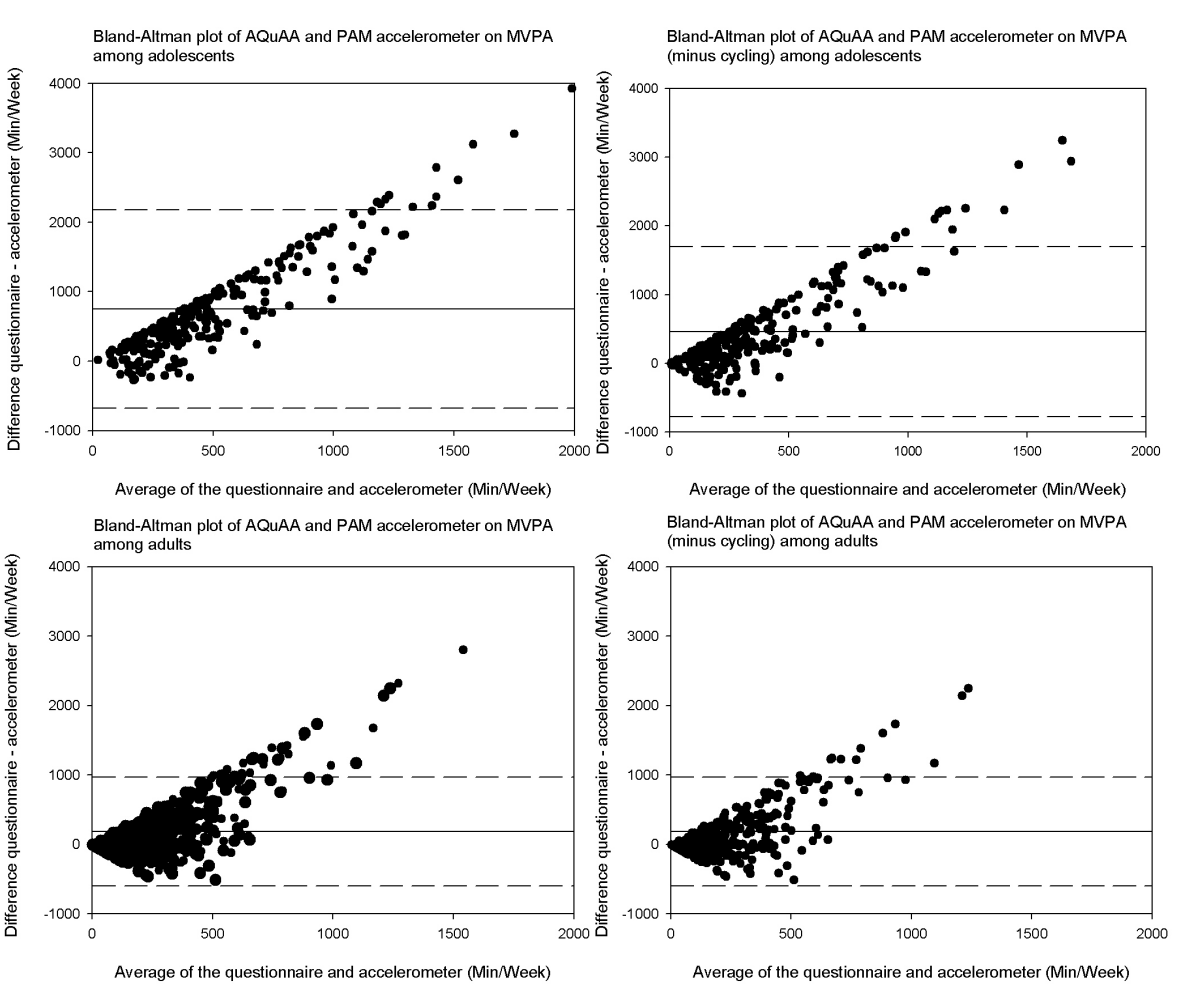
**Bland-Altman plots of moderate-to-vigorous physical activity (min/wk) assessed by AQuAA (with and without reported cycling) and PAM accelerometer among adolescents and adults**.

Median with 25^th ^and 75^th ^percentiles of MPA, VPA, MVPA (including and excluding reported minutes spent on cycling) are displayed for the different subgroups of adolescents and adults in additional file 2 and additional file 3, respectively. Additional file 2 and additional file 3 present the minutes of MPA, VPA and MVPA assessed by the accelerometer expressed as percentage of the self-reported minutes. Time spent on MPA, VPA and MVPA according to questionnaire was practically always higher than recorded by the accelerometer and differed significantly (p < 0.05) in all subgroups of adolescents and adults. The Bland-Altman plots of these data on MPA, VPA and MVPA, in figure 2 and figure 3, show overall higher readings from the questionnaire than the accelerometer, but considerable variation in the individual differences between the questionnaire and accelerometer estimates.

Among adolescents, in a multivariate model level of education remained a statistically significant predictor of disagreement in MPA (304 min/wk) and MVPA (361 min/wk). For VPA, gender was a significant predictor (135 min/wk). Among adults, overweight was a significant predictor of disagreement in VPA (78 min/wk) and MVPA (114 min/wk) between AQuAA and PAM.

### Gender

The accelerometer measurement showed that adolescents boys were more active on MPA compared to adolescents girls (112 vs. 79 minutes, *p *= 0.04), however the opposite was found for the self-report (503 vs. 532 minutes, *p *= 0.03). Adolescent boys also reported significantly more minutes of VPA (180 vs. 0 minutes, p < 0.001) than girls, but this difference was again not confirmed by the accelerometer data (13 vs. 14 minutes, *p *= 0.74). Among adults, self-reported and measured MPA and VPA did not differ significantly between men and women.

### Education

According to the questionnaire, adolescents with a low educational level were less active on MPA (360 vs. 565 minutes, *p *= 0.002) and VPA (0 vs. 120 minutes, *p *= 0.012) compared to higher educated adolescents. However, the accelerometer measurement showed for both MPA (200 vs. 51 minutes, p < 0.001) and VPA (54 vs. 7 minutes, p < 0.001) the opposite. Among adults, no significant differences in reported or measured MPA and VPA between educational levels were found.

### Gender combined with education

**Figure 4:**
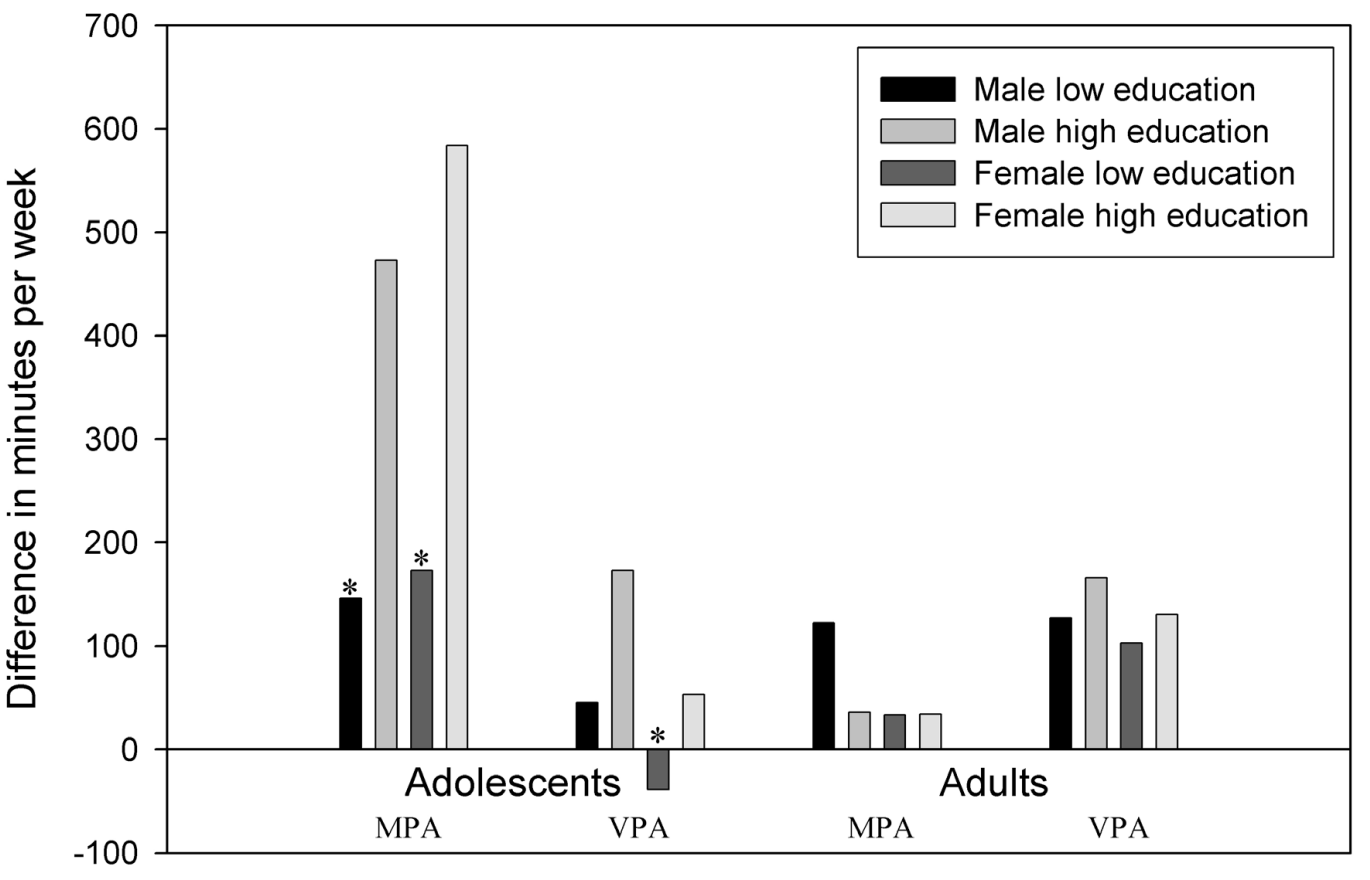
**Disagreement between self-report and objective assessment of moderate and vigorous physical activity (min/wk) in subgroups of age, gender and education**. Note. MPA: moderate physical activity; VPA: vigorous physical activity. * Significant different (p < 0.05) from the peers of the same gender.

Figure 4 shows the disagreement in MPA and VPA among subgroups of gender and level of education for adolescents and adults. According to the questionnaire, lower educated girls spent less time on MPA (350 vs. 625 minutes, *p *= 0.03) and VPA (0 vs. 60 minutes, *p *= 0.05) than their higher educated peers. In boys a similar pattern between low and high-educated subjects is seen in MPA (405 vs. 540 minutes, *p *= 0.19) and VPA (140 vs. 180 minutes, *p *= 0.87). However, the accelerometer data for both boys and girls in MPA and VPA show the opposite (p < 0.001).

Among adults, similar results for subgroups were found for MPA for both measures, with exception of the lower educated men who reported two times more minutes of MPA compared to the accelerometer. For VPA, the reported minutes (range 120 to 180 minutes) were higher than the recorded minutes by the accelerometer (range 9 to 28 minutes) between subgroups.

### Weight status

According to the questionnaire normal weight adolescents were more active in MPA and VPA than their overweight peers (non-significant), but according to the accelerometer normal weight adolescents were less active in MPA (81 vs. 162 minutes, *p *= 0.008) and VPA (12 vs. 29 minutes, *p *= 0.05). On the contrary, overweight adults reported more VPA than those with normal weight (185 vs. 128 minutes, *p *= 0.04), but this was not confirmed by the accelerometer (9 vs. 18 minutes, *p *= 0.25).

### MVPA with and without reported cycling

Multivariate regression analyses showed that minutes spent on cycling were a significant contributor to the disagreement between both PA measures among adolescents and adults.

**Figure 5:**
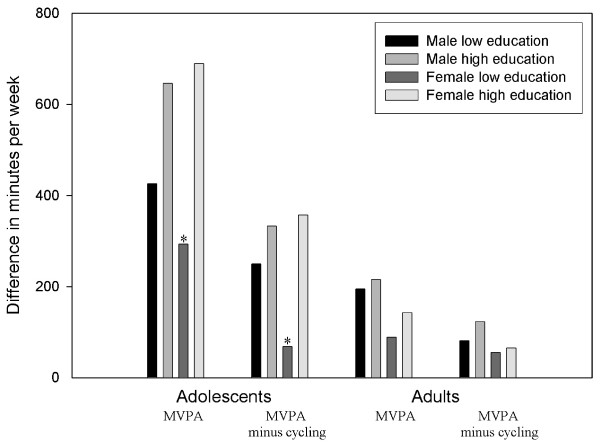
**Disagreement between self-report (with and without reported cycling) and objective assessment of moderate-to- vigorous physical activity (min/wk) in subgroups of age, gender and education**. Note. MVPA: moderate-to-vigorous physical activity. * Significant different (p < 0.05) from the peers of the same gender.

According to the questionnaire adolescents spent 230 minutes per week (median) on cycling. Moreover, adolescents with a high educational level spent more time cycling (median 250 vs. 135 minutes per week, *p *= 0.001) compared to lower educated adolescents. Among adults, median time spent on cycling was 40 minutes. Additional file 2 and additional file 3 show the relative disagreement of both PA measures in MVPA with and without reported cycling. As anticipated, disagreement of MVPA without cycling was overall smaller than MVPA with cycling. Figure 5 shows the disagreement in MVPA with and without reported cycling among subgroups of gender and level of education for adolescents and adults. In the total group and subgroups of adolescents and adults, PA patterns were similar for both MVPA.

## Discussion

In our study we found that 7 to 22% of the time spent on MPA and VPA reported by adolescent boys and girls was objectively confirmed by the PAM accelerometer. Contrary to what was observed among adults, the disagreement among adolescents with a high educational level was greater than adolescents with a low educational level. This disagreement was greater among girls than boys. Among adults there was generally, better agreement between both measures with regard to MPA than among adolescents. However, there was also a strong disagreement with regard to VPA, particularly among overweight adults.

Neither the questionnaire nor the PAM accelerometer is a gold standard for measuring PA and disagreement between the instruments is a result of weaknesses in both instruments. Therefore, we can only make a relative comparison between the instruments.

Since self-reported PA is prone to misreporting and the correlations between the AQuAA questionnaire and accelerometer were low, results should be interpreted with caution. Based on the fair reproducibility and the low validity of the AQuAA we may conclude that the disagreements between self-report and accelerometer found in our study are relevant, however the magnitude of this disagreement remains unclear.

Potential misreporting in the questionnaire may be the result of the fact that most daily activities are intermittent and may involve significant breaks or rest periods. This may lead to inaccurate recall and significant overestimation of time spent on daily activities [19,33-35]. The higher levels of PA reported in the questionnaire may also be the result of misreporting by subjects that did not report over the last week, but over the two-week period that the accelerometer was worn.

Measurement errors of the accelerometer may also have led to the disagreement between both instruments. Our study showed that cycling was a significant contributor to this disagreement. Analyses without reported time spent on cycling showed that the difference in time spent on MVPA between both measures only remained significant in subgroups of education among adolescents. Likewise, disagreement between the questionnaire and the accelerometer may also be partly due to not wearing the accelerometer during playing sports however this adherence did not differ between the subgroups.

Continuous behaviors with a higher perceived intensity than objectively measured could also contribute to the disagreement between the two PA methods. Misclassification of activity intensity may be related to the application of MET cut-offs to both self-report and accelerometer data. For instance, when a light activity is classified as moderate by respondents to the questionnaire, misclassification is introduced. Since most of the respondents are not familiar with the MET metric and can not differentiate between the 3 MET and 4 MET, we have provided example activities (based on the compendium of Ainsworth [36]) along with the questions to enable the respondent to make an estimation of the activity intensity.

Few studies have examined and compared the variation between an objective and a subjective PA measure [13,37]. An earlier study of Sallis et al [13], showed ethnic and gender differences among adolescents in disagreement of PA assessments.

In our study, the disagreement with respect to MPA was greatest for higher educated adolescents, especially in girls. Possibly higher educated adolescents reported more social desirable answers or they were involved in different moderate intensity activities than their peers which caused greater disagreement.

In adults, there was greater disagreement between the two measures among overweight adults with respect to VPA. Although all respondents experience difficulty estimating the activity intensity and the duration of activity, overweight adults may rate an activity more easily as VPA than normal weight adults. This finding for VPA and MVPA in overweight people is also found in other studies [38,39] and is relevant information by interpreting PA trends in subgroups of weight status. So, using questionnaires on a population level to assess PA levels may underestimate the difference in VPA between people with a normal weight and people with overweight.

A considerable strength of the study is the high compliance for wearing the accelerometer. Applying the Spearman-Brown prophecy formula on the separate age-groups, there can be concluded that a minimum of 5 days of measurement was required to achieve a reliability level of 80% [40]. In our study, 90% of the adolescents and 92% of the adults wore the accelerometer for at least five out of the seven days in the last week. This compliance is high compared with previous studies (range 62–75% [13,41]).

Subjects were recruited from different schools and companies in the surroundings of Amsterdam, which favors the generalizability of the study results. However, since our findings are based on one particular accelerometer and one questionnaire, the results cannot be generalized to other accelerometers and questionnaires. Also, since, our study participants also consented to participate in an intervention study aimed at increasing PA (see method section), our study sample may be more health conscious than the general population. Both aspects may limit the generalizability of the findings.

In summary, we observed considerable disagreement in time spent on MPA and VPA measured by an objective measure compared to a subjective measure in adolescents. Adolescent girls with a high educational level extremely over-reported MPA and boys over-report VPA relative to accelerometer registered time. In adults there was moderate agreement between both measurement methods with regard to MPA but not to VPA. Disagreement on time spent on MPA was largest among men with a low educational level and disagreement on time spent on VPA was largest among overweight adults. However, since both measures are not a gold standard, it is not possible to determine the exact size of the misclassification of the instruments.

## Conclusion

This study highlighted important differences between questionnaire and accelerometer in PA estimates, both among adolescents and adults. On average, questionnaires produced substantially higher estimates of PA participation. A notable concern was observed among adolescents, where readings from the questionnaire were often opposite to readings from the accelerometer among subgroups of gender and educational level. There is a clear need in advanced valid assessments of MPA and VPA in adolescents.

## Abbreviations

PA: physical activity; MPA: moderate intensity physical activity; VPA: vigorous intensity physical activity; MVPA: moderate-to-vigorous physical activity.

## Competing interests

The authors declare that they have no competing interests.

## Authors' contributions

SMS was the primary author responsible for conducting the research, statistical analysis, interpretation of data, and drafting and revising the manuscript. MCAP and AJS designed the study, participated in the coordination of the study and in writing the article. WvM and JCS have been involved in the design of the study and drafting the manuscript. All authors read and approved the manuscript.

## Supplementary Material

Additional file 1 **Activity Questionnaire for Adults and Adolescents (AQuAA)** Click here for file

Additional file 2 **Table S2.** Median (25^th ^and 75^th ^percentiles) physical activity (min/wk) assessed by questionnaire (AQuAA) and accelerometer (PAM) among adolescents. Click here for file

Additional file 3 **Table S3.** Median (25^th ^and 75^th ^percentiles) physical activity (min/wk) assessed by questionnaire (AQuAA) and accelerometer (PAM) among adults. Click here for file
