# Supplementary material for: Disagreement in physical activity assessed by accelerometer and self-report in subgroups of age, gender, education and weight status
Source: Int J Behav Nutr Phys Act. 2009 Mar 25;6:17. doi: 10.1186/1479-5868-6-17 (PMC2670257; doi:10.1186/1479-5868-6-17)
Supplement: Additional file 2 — Table S2. Median (25th and 75th percentiles) physical activity (min/wk) assessed by questionnaire (AQuAA) and accelerometer (PAM) among adolescents. [file 1479-5868-6-17-S2.doc]

**Table S2. Median (25^th^ and 75^th^ percentiles) physical activity (min/wk)** assessed by questionnaire (AQuAA) and accelerometer (PAM) among adolescents.

| **Variable** | **Method** |  | **Total** | **Male** | **Female** | **p** | **Low Educated** | **High**  **Educated** | **p** | **Normal Weight** | **Overweight** | **p** |
| --- | --- | --- | --- | --- | --- | --- | --- | --- | --- | --- | --- | --- |
| **MPA** | **AQuAA** | **%** | **100** | **100** | **100** |  | **100** | **100** |  | **100** | **100** |  |
|  |  | **min/wk** | 528 (276-896) | 503 (279-936) | 532 (274-870) | .93 | 360 (120-787) | 565 (360-928) | .002 | 530 (280-883) | 480 (170-1009) | .71 |
|  | **PAM** | **% *** | **18** | **22** | **15** |  | **56** | **9** |  | **15** | **34** |  |
|  |  | **min/wk** | 93 (30-193) | 112 (38-233) | 79 (25-161) | .04 | 200 (120-255) | 51 (21-120) | .001 | 81 (28-178) | 162 (97-259) | .008 |
| **VPA** | **AQuAA** | **%** | **100** | **100** | **100** |  | **100** | **100** |  | **100** | **100** |  |
|  |  | **min/wk** | 60 (0-300) | 180 (0-460) | 0 (0-270) | .001 | 0 (0-268) | 120 (0-330) | .01 | 60 (0-325) | 0 (0-208) | .18 |
|  | **PAM** | **% *** | **23** | **7** | **14/0** |  | **54/0** | **6** |  | **20** | **29/0** |  |
|  |  | **min/wk** | 14 (3-50) | 13 (3-58) | 14 (3-45) | .74 | 54 (16-97) | 7 (2-28) | .001 | 12 (3-48) | 29 (9-65) | .05 |
| **MVPA** | **AQuAA** | **%** | **100** | **100** | **100** |  | **100** | **100** |  | **100** | **100** |  |
|  |  | **min/wk** | 723 (408-1234) | 775 (460-1519) | 653 (366-1074) | .02 | 510 (195-1200) | 760 (475-1260) | .02 | 740 (443-1249) | 553 (268-1044) | .31 |
|  | **PAM** | **% *** | **15** | **16** | **15** |  | **43** | **8** |  | **13** | **30** |  |
|  |  | **min/wk** | 108 (34-220) | 122 (35-289) | 100 (34-206) | .15 | 217 (129-307) | 61 (25-139) | .001 | 96 (33-216) | 166 (110-262) | .04 |
| **MVPA** | **AQuAA** | **%** | **100** | **100** | **100** |  | **100** | **100** |  | **100** | **100** |  |
| **(excluding cycling)** |  | **min/wk** | 460 (103-1148) | 480 (203-1144) | 363 (180-660) | .07 | 310 (118-750) | 475 (235-783) | .03 | 420 (185-783) | 420 (170-690) | .70 |
|  | **PAM** | **% *** | **23** | **25** | **28** |  | 70 | **13** |  | **23** | **40** |  |

* The minutes of activity assessed by the accelerometer expressed as percentage of the minutes of activity by questionnaire.

Note: MPA: moderate intensity physical activity, VPA: vigorous intensity physical activity, MVPA: moderate-to-vigorous intensity physical activity.
