# Supplementary material for: Disagreement in physical activity assessed by accelerometer and self-report in subgroups of age, gender, education and weight status
Source: Int J Behav Nutr Phys Act. 2009 Mar 25;6:17. doi: 10.1186/1479-5868-6-17 (PMC2670257; doi:10.1186/1479-5868-6-17)
Supplement: Additional file 3 — Table S3. Median (25th and 75th percentiles) physical activity (min/wk) assessed by questionnaire (AQuAA) and accelerometer (PAM) among adults. [file 1479-5868-6-17-S3.doc]

**Table S3. Median (25^th^ and 75^th^ percentiles) physical activity (min/wk)** assessed by questionnaire (AQuAA) and accelerometer (PAM) among adults.

| **Variable** | **Method** |  | **Total** | **Male** | **Female** | **p** | **Low Educated** | **High**  **Educated** | **p** | **Normal Weight** | **Overweight** | **p** |
| --- | --- | --- | --- | --- | --- | --- | --- | --- | --- | --- | --- | --- |
| **MPA** | **AQuAA** | **%** | **100** | **100** | **100** |  | **100** | **100** |  | **100** | **100** |  |
|  |  | **min/wk** | 120 (0-300) | 120 (6-360) | 120 (0-286) | .40 | 150 (36-350) | 120 (0-300) | .22 | 120 (0-265) | 150 (6-360) | .29 |
|  | **PAM** | **% *** | **70** | **68** | **70** |  | **56** | **70** |  | **70** | **57** |  |
|  |  | **min/wk** | 84 (30-152) | 82 (30-162) | 84 (30-148) | .75 | 84 (21-147) | 84 (34-158) | .55 | 84 (36-149) | 86 (24-170) | .85 |
| **VPA** | **AQuAA** | **%** | **100** | **100** | **100** |  | **100** | **100** |  | **100** | **100** |  |
|  |  | **min/wk** | 155 (60-308) | 0 (0-270) | 175 (60-300) | .63 | 135 (60-300) | 170 (59-321) | .62 | 128 (30-300) | 185 (86-344) | .04 |
|  | **PAM** | **% *** | **9** | **15/0** | **7** |  | **13** | **8** |  | **14** | **5** |  |
|  |  | **min/wk** | 14 (0-68) | 15 (1-75) | 12 (0-55) | .32 | 18 (0-68) | 13 (0-68) | .58 | 18 (1-79) | 9 (0-56) | .25 |
| **MVPA** | **AQuAA** | **%** | **100** | **100** | **100** |  | **100** | **100** |  | **100** | **100** |  |
|  |  | **min/wk** | 340 (171-613) | 360 (201-660) | 330 (150-580) | .18 | 300 (161-660) | 360 (178-593) | .67 | 305 (170-530) | 390 (180-731) | .07 |
|  | **PAM** | **% *** | **42** | **39** | **44** |  | **45** | **41** |  | **49** | **36** |  |
|  |  | **min/wk** | 144 (77-246) | 142 (77-250) | 144 (76-245) | .93 | 134 (72-257) | 149 (78-240) | .65 | 150 (83-240) | 140 (64-256) | .99 |
| **MVPA** | **AQuAA** | **%** | **100** | **100** | **100** |  | **100** | **100** |  | **100** | **100** |  |
| **(excluding cycling)** |  | **min/wk** | 248 (120-510) | 293 (120-540) | 210 (105-490) | .11 | 240 (120-505) | 258 (118-510) | .98 | 240 (120-465) | 300 (124-600) | .11 |
|  | **PAM** | **% *** | **58** | **48** | **69** |  | **56** | **58** |  | **63** | **47** |  |

* The minutes of activity assessed by the accelerometer expressed as percentage of the minutes of activity by questionnaire.

Note: MPA: moderate intensity physical activity, VPA: vigorous intensity physical activity, MVPA: moderate-to-vigorous intensity physical activity
